# A Case Report of Prilocaine-Induced Methemoglobinemia after Liposuction Procedure

**DOI:** 10.1155/2015/282347

**Published:** 2015-06-23

**Authors:** Birdal Yildirim, Ulku Karagoz, Ethem Acar, Halil Beydilli, Emine Nese Yeniceri, Ozgur Tanriverdi, Omer Dogan Alatas, Şükrü Kasap

**Affiliations:** ^1^Department of Emergency Medicine, Muğla Sıtkı Koçman University Medical Faculty, Orhaniye Mahallesi Haluk Ozsoy Caddesi, 48000 Mugla, Turkey; ^2^Emergency Clinic, Muğla Sıtkı Koçman University Training and Investigation Hospital, Orhaniye Mahallesi Haluk Ozsoy Caddesi, 48000 Mugla, Turkey; ^3^Department of Family Medicine, Muğla Sıtkı Koçman University Medical Faculty, Orhaniye Mahallesi Haluk Ozsoy Caddesi, 48000 Mugla, Turkey; ^4^Department of Internal Medicine and Medical Oncology, Muğla Sıtkı Koçman University Medical Faculty, Orhaniye Mahallesi Haluk Ozsoy Caddesi, 48000 Mugla, Turkey; ^5^Clinic of Plastic and Reconstructive Surgery, Muğla Sıtkı Koçman University Training and Investigation Hospital, Orhaniye Mahallesi Haluk Ozsoy Caddesi, 48000 Mugla, Turkey

## Abstract

Prilocaine-induced methemoglobinemia is a rarely seen condition. In this paper, a case is presented with methemoglobinemia developed secondary to prilocaine use in a liposuction procedure, and the importance of this rarely seen condition is emphasized. A 20-year-old female patient presented with complaints of prostration, lassitude, shivering, shortness of breath, and cyanosis. It was learned that the patient underwent nearly 1000 mg prilocaine infiltration 8 hours priorly during a liposuction procedure. At admission, her blood pressure (130/80 mmHg), pulse rate (140 bpm), body temperature (36°C), and respiratory rate (40/min) were recorded. The patient had marked acrocyanosis. The arterial blood gas methemoglobin level was measured as 40%. The patient received oxygen therapy with a mask and was administered vitamin C in normal saline (500 mg tid), N-acetylcysteine (300 mg tid), and 50 mg 10% methylene blue in the intensive care unit of the internal medicine department. Methemoglobin level dropped down to 2% after her treatment with methylene blue and she was clinically cured and discharged 2 days later. Emergency service physicians should remember to consider methemoglobinemia when making a differential diagnosis between dyspnea and cyanosis developing after prilocaine infiltration performed for liposuctions in the adult age group.

## 1. Introduction

Methemoglobinemia is defined as an increase in the blood methemoglobin level, and it is an important cause of cyanosis [1]. When ferrous (Fe^2+^) hemoglobin oxidizes iron and transforms it into a ferric (Fe^3+^) cation, methemoglobin forms. The methemoglobin level is held at low levels via the reductizing cytochrome b5 reductase enzyme (NADH-methemoglobin reductase) levels in erythrocytes [1]. Indeed, methemoglobin is not functional in contrast with oxygen-carrying hemoglobin, and conditions with increased blood methemoglobin levels convey clinical importance [2]. The most important clinical symptom of methemoglobinemia is cyanosis, and it should be considered in particular in cyanotic patients with normal cardiovascular and pulmonary functions [2]. In mild cases, clinical signs and symptoms may not be observed; however, severe cases may present with cyanosis, tachypnea, hypotension, tachycardia, and confusion. Advanced cases may be fatal [2].

The pathophysiology of methemoglobinemia involves an imbalance between oxidation and reduction [1, 2]. It may emerge as a hereditary or acquired condition [2]. The most important cause of acquired methemoglobinemia is exposure of healthy individuals to drugs or chemical substances [2]. Such chemicals as nitrite, nitrate, aniline, and benzene compounds and drugs like sulfonamides, dapsone, phenacetin, primaquine, and benzocaine make up the most important agents [2, 3]. Prilocaine, an amide compound that is frequently used as a local anesthetic, can also cause methemoglobinemia [3].

Based on a literature review, cases with prilocaine-induced methemoglobinemia are frequently seen in neonatal and early pediatric ages [4, 5]. Prilocaine-induced methemoglobinemia during liposuction is rarely seen. In this paper, in consideration of the rarity of this condition, a severe case of prilocaine-induced methemoglobinemia in a patient who underwent regional liposuction is presented.

## 2. Case Presentation

A 20-year-old female patient without any previous systemic disease applied to this emergency service with complaints of shivering, prostration, lassitude, fatigue, shortness of breath, and cyanosis of the lips. The general health state of the patient was deteriorated. An inspection of the patient revealed a tachypneic pulse and cyanotic lips. The patient's blood pressure (130/80 mm/Hg), respiratory rate (40/min), pulse rate (rhythmic, 140 bpm), body temperature (36°C), and oxygen saturation (SpO_2_, 82%) were measured with normal pulmonary and cardiovascular examination findings. Her chest X-ray and electrocardiographic examination results were within normal limits. The whole blood cell count and blood biochemistry did not yield any abnormal results, which would explain the clinical health state and metabolic, hematological, infective, renal, and hepatic diseases of the patient. Similarly, no respiratory or cardiovascular abnormality that could account for her actual clinical health state was detected. With an arterial blood gas pH, 7.46, pCO_2_, 31.1 mm Hg, pO_2_, 83.3 mm Hg, HCO_3_, 20 mEq/L, lactate, 3.6 mmol/L, and methemoglobin, 40%, the patient was considered to have methemoglobinemia. Despite the initiation of a saline infusion (100 mL/hr) and oxygen delivery with a mask at a rate of 10 mL/kg, her cyanosis persisted, and the patient was monitored in the intensive care unit of the department of internal medicine.

When the patient was stabilized, it was learned from her detailed anamnesis that she had undergone liposuction from her medial aspects of both femoral regions eight hours prior to the onset of her complaints. A total of 1000 mg prilocaine was used during the liposuction procedure, and 100–200 mg of the drug might be retained within the procedural site. Her treatment was continued with a vitamin C (3 × 500 mg) infusion in normal saline, N-acetylcysteine (3 × 300 mg), and 50 mg 10% methylene blue. Her methemoglobin level dropped to 2%, and her clinical findings improved. She was discharged two days after her admission into the intensive care unit.

## 3. Discussion

In this case report, in consideration of its rarity, an adult patient who consulted emergency services with clinical manifestations of severe methemoglobinemia which developed after the application of prilocaine during a regional liposuction is presented.

Methemoglobinemia can develop under the influence of hereditary and acquired factors [2]. Many drugs and chemical substances are known to induce acquired methemoglobinemia. The local anesthetic prilocaine is among these methemoglobinemia-inducing drugs [2, 6].

As a local anesthetic drug, the therapeutic dose of prilocaine has been reported as 1-2 mg/kg, and, in cases with methemoglobinemia emerging at its therapeutic doses, cyanosis cannot be observed [7]. The maximum safe dose of prilocaine is 8 mg/kg, while the daily maximum dose is 600 mg [8]. However, increasingly higher doses have been used during liposuction procedures [9]. It has been reported that, despite higher doses of prilocaine being used in the tumescent liposuction method, the risk of methemoglobinemia is lower, which indicates the safety of this liposuction method [9]. In a study of 25 cases in which patients had undergone liposuction with this technique, methemoglobinemia had not developed [10]. In the tumescent infiltrative liposuction technique, a substantial amount of local anesthetic infiltrated subcutaneously is removed from the body via the aspiration method [11]. The biological half-life of prilocaine is 55 minutes, and the time required for the development of methemoglobinemia is 20–60 minutes [8]. In this case, 1000 mg prilocaine was infiltrated and nearly 800–900 mg of the drug was aspirated. A total of 100–200 mg was aspirated by the body cavities. It is believed that, despite the use of lower than toxic doses of prilocaine, the prolongation of the procedure for 6–8 hours induced the development of methemoglobinemia.

Methemoglobinemia should be taken into consideration in the differential diagnosis of patients with normal circulatory and respiratory system findings who consult with cyanosis [2]. Under physiological conditions, methemoglobin constitutes 1% of Hb and does not exceed more than 2-3% of Hb [1]. However, a derangement of the balance between oxidation and reduction can increase the prilocaine concentration above physiologic levels [2]. Exposure to some oxidant substances can induce methemoglobinemia even in healthy individuals. However, in healthy individuals, the increased methemoglobin concentration is lowered to normal levels by means of the cytochrome b5 reductase enzyme found in red blood cells [1, 3, 6]. In some cases, the compensation mechanism does not function properly, and increasing levels of methemoglobin cannot transport O_2_, which shifts the hemoglobin-oxygen dissociation curve to the left, thereby complicating the delivery of oxygen to the tissues [1, 3, 6].

Mild cases can be asymptomatic, but, in severe cases, cyanosis, tachypnea, tachycardia, hypotension, confusion, or even death may be observed [4]. In cases with methemoglobinemia, varying degrees of cyanosis can be detected which are associated with blood methemoglobin levels [3, 6]. When blood methemoglobin levels exceed 10%, peripheral cyanosis becomes apparent, and, in cases with methemoglobin levels of ≥35%, tissue hypoxia and diffuse cyanosis are seen. When methemoglobin levels approach 70%, the patient enters a coma and if untreated may die [1, 3, 6].

The patient who received prilocaine during a regional liposuction had a methemoglobin level of 40% at admission and presented clinically with fatigue, shivering, diffuse perioral and peripheral cyanosis, tachypnea, and tachycardia. The methemoglobinemia level of this patient and the clinical findings were compatible with the literature findings.

During the first months of life, a transient deficiency of cytochrome b5 reductase enzyme activity predisposes newborns and infants to methemoglobinemia [1]. However, since during the first 3 months of life the cytochrome b5 reductase activity is 50% of the level found in adults, even with therapeutic doses of prilocaine, methemoglobinemia can develop [7]. Therefore, many cases with methemoglobinemia can be found in the literature which have been encountered during surgical procedures performed on newborns with local anesthesia [4, 5, 12].

The first measure to be applied after the development of methemoglobinemia following an exposure to a chemical agent or drug is the prevention of exposure to this substance. Patients with levels of methemoglobin over 20% can improve spontaneously, and it must be remembered that, above this level, clinical manifestations can worsen [13]. As treatment alternatives, methylene blue, ascorbic acid, and riboflavin are recommended for these patients. The mortality risk is higher in cases with methemoglobin levels of ≥70%; hyperbaric oxygen therapy is recommended for these patients [14]. However, methylene blue is the first treatment alternative for all cases. Animal and* in vitro* human experiments have demonstrated that ascorbic acid decreases the methemoglobin levels through a nonenzymatic process [14]. Additionally, in previous* in vitro* studies, N-acetylcysteine has been reported to be an important alternative approach in the treatment of nitric oxide related methemoglobinemia [15, 16]. However, N-acetylcysteine was shown to have no effect on human volunteers in a study conducted by Tanen et al. [17]. Similarly, in a study carried out by Dötsch et al. [18], effects of treatment with methylene blue, riboflavin, and N-acetylcysteine were compared in patients with methemoglobinemia but N-acetylcysteine and low-dose riboflavin were found not to change methemoglobin formation. Therefore, N-acetylcysteine has not yet found a place in clinical practice as treatment for methemoglobinemia [18, 19]. However, it should be kept in mind that methylene blue is contraindicated in patients with glucose-6-phosphate dehydrogenase deficiency whose methemoglobinemia, paradoxically, may worsen [19].

Since this patient's level of methemoglobinemia was 40%, systemic toxic symptoms were in the foreground, and intravenous infusion of methylene blue at a rate of 1-2 mg/kg/5 min was initiated when the antioxidant therapy failed to elicit an adequate response.

In conclusion, methemoglobinemia should be considered in patients with incompatible oxygen saturation and pO_2_ results who developed cyanosis following minimally invasive surgical interventions. With perfect history taking and appropriate differential diagnoses, methemoglobinemia treatment can be improved without giving rise to the deterioration of the clinical picture.
